# Analysis of the Clinical Course of Experimental Infection with Highly Pathogenic African Swine Fever Strain, Isolated from an Outbreak in Poland. Aspects Related to the Disease Suspicion at the Farm Level

**DOI:** 10.3390/pathogens9030237

**Published:** 2020-03-22

**Authors:** Marek Walczak, Jacek Żmudzki, Natalia Mazur-Panasiuk, Małgorzata Juszkiewicz, Grzegorz Woźniakowski

**Affiliations:** Department of Swine Diseases, National Veterinary Research Institute, Partyzantów 57 Avenue, 24-100 Puławy, Poland; jaca@piwet.pulawy.pl (J.Ż.); natalia.mazur@piwet.pulawy.pl (N.M.-P.); malgorzata.juszkiewicz@piwet.pulawy.pl (M.J.); grzegorz.wozniakowski@piwet.pulawy.pl (G.W.)

**Keywords:** ASF, experimental infection, ASFV, clinical course, veterinary diagnosis

## Abstract

This paper was aimed to characterize clinical signs and pathomorphological lesions in twenty-two pigs, infected intranasally by different doses of African swine fever virus (Pol18_28298_O111), isolated during the outbreak in a pig farm that occurred in Eastern Poland throughout 2018. This article also attempts to indicate risk, related to virus load and shedding, and present possible difficulties with proper disease recognition at the farm level. The results revealed that even a very low dose (5 HAU) may initiate the infection. Various forms of the disease (acute, subacute, and chronic), mainly with prodromal clinical signs like fever, apathy, and reduced feed intake were observed. The most frequently observed lesions (82%) were: hyperemia and enlargement of lymph nodes and splenomegaly. The minimal incubation period was estimated at five days post-infection (dpi). Mortality ranged from 80–100%. Two pigs survived the infection. Some viremic animals presented delayed fever. In some cases, the fever was not detectable. Shortly after viremia, the virus was secreted ion the urine, feces, and saliva. The highest levels of virus were found in the internal organs and blood; however in the case of one pig (chronic form), viral DNA was not detected in the spleen, liver, bone marrow, and brain. Veterinary diagnosis may be difficult, and the final results should always be based on laboratory investigations.

## 1. Introduction

African swine fever (ASF), caused by the African swine fever virus (ASFV), remains one of the most dangerous infectious diseases of pigs and presents an urgent problem, mainly due to its impact on the global economy and welfare of pigs around the world. Today, in the light of numerous failures in developing an effective vaccine against ASF, prevention is mainly based on biosecurity measures together with the efficient work of veterinary services [1,2,3]. 

ASF reached Europe (Georgia) for the second time in 2007 [4,5]. Presently, the disease affected most of Eastern Europe and several Asian countries and started to pose a threat to countries of Western Europe [6]. The genetic investigation of the Georgia 2007 strain classified the virus isolated to genotype II of the ASFV [5]. So far, several studies of “Georgia–like” strains, isolated in different areas of Europe and Asia, confirmed their biological diversity. Clinical signs of the examined isolates ranged from hemorrhagic-like fever to subclinical form of the disease, where some animals stayed apparently healthy [7,8]. 

The understanding of the biological properties of different ASFV strains plays a major role in updating knowledge about the possible clinical or pathomorphological course of the disease. The Veterinary Services take advantage of these observations in case of an ASF outbreak. Moreover, identified in the future, low virulent, naturally attenuated isolates may be crucial for vaccine development [3,9]. Therefore, studies of the biological properties of ASFV strains are still necessary. 

Diagnostic tools used in routine monitoring of the disease presents high specificity and sensitivity; however, confirmation in the laboratory may be time-consuming. The Veterinary Services, which are the supervisors of animal health within pig farms, should be particularly sensitive for any potential clinical signs of ASF and must be aware of possible scenarios of the disease course [10]. Veterinarians together with staff responsible for animal facilities, are the first who may contact affected animals. Their awareness about proper procedures of diagnosis, handling, and disposal of suspicious animals, as well as knowledge of the actual epidemiology may play a key role in prevention and minimizing the consequences of a potential outbreak [4].

The aim of this study was to present possible clinical signs and pathomorphological lesions in the course of the ASF, caused by virus isolated during an outbreak in a pig farm in Poland (2018). This article also attempts to indicate the risk related to virus load and shedding and presents possible difficulties with a proper initial diagnosis of the disease at the farm level.

## 2. Results

### 2.1. Clinical Findings

#### 2.1.1. Group I—1000 Hemadsorbing Units (HAU)

**Figure 1:**
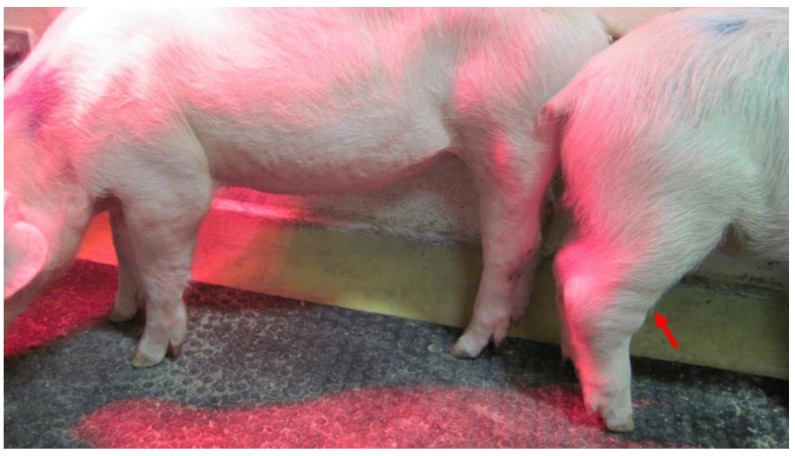
Chronic form of African swine fever (ASF). The arrow indicates swollen joints.

Ordinary, typical for ASF, but non-specific clinical signs like fever, body paleness, listlessness, and reduced feed intake, leading to animals’ death were recorded during the experiment. At the beginning, the acute (fever, death within 3–4 days of life with viremia, a sharp increase of the virus load in the blood (n = 3)) and subacute (moderate or high fever and other clinical signs, 5–8 days of life with viremia, moderate and progressing increase of virus load value in the blood (n = 4)) form of the disease have been observed. Pig #8 survived the infection and developed a chronic form of the disease (varied, clearly visible clinical signs, moderate fever, constant, low virus load value in the blood (n = 1)) presenting additionally other clinical signs like joint swelling (Figure 1) and minor breathing disorders. Interestingly in the case of this pig, a partial remission of the observed clinical signs was recorded after 29 dpi. The pig was euthanized at 32 dpi.

It is noteworthy, that in the acute form of the disease no other clinical signs, except high fever (>41.5 °C) were recorded; moreover, one pig was still interested in feed, despite the detected viremia and high fever. 

**Figure 2:**
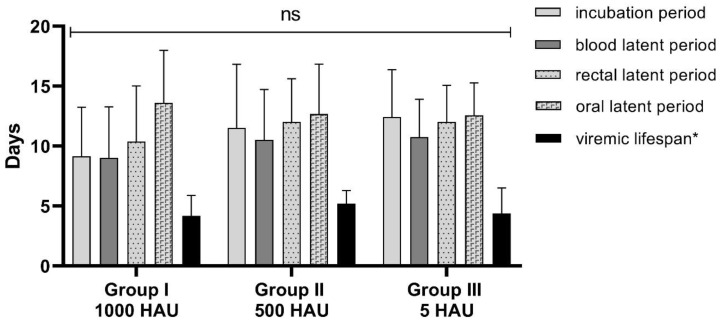
The average timespan between the first day of infection and: detection of fever (incubation period), ASFV DNA detection in blood, rectal, or oral specimens (latent period). The average lifespan of animals with detected viremia; *—relevant only for the subacute and acute form of the disease; ns—not significant.

The minimum incubation period was estimated as five days, maximum as 16 days with an average of 9 (± 4) days. The average lifespan with the viremia was estimated as 4 (± 2) days (pig with chronic form was not included in this analysis) (Figure 2). The mortality in this Group reached nearly 90% (seven out of 8 pigs died due to infection). 

#### 2.1.2. Group II—500 HAU

The acute (n = 2) and subacute (n = 3) form of the disease were noticed. Fever, body paleness, listlessness, and reduced feed intake were observed similarly to Group I. Extraordinary clinical signs like convulsions were observed in the case of two animals that have been euthanized. Pig #14 developed a chronic form of the disease, contrary to Pig #8 (Group I), without joint swelling, breathing disorders, or other clinical signs, but with moderate fever. This pig was euthanized at 24 dpi.

Similarly to the other groups, the minimum incubation period was estimated as five days; however, the maximum was the highest, estimated as 20 days (Pig #14—with a chronic form of the disease). The average incubation period was estimated as 12 (± 5) days. The average lifespan with viremia was estimated as 5 (± 1) days (pig with chronic form was not included in this analysis) (Figure 2). Five out of six pigs died due to infection (mortality reached nearly 80%).

#### 2.1.3. Group III—5 HAU

Correspondingly to Group I, acute (n = 4) and subacute (n = 4) forms of disease were noticed with very similar clinical signs. However, no survivors and no chronic form of the disease were observed during the experiment in this Group. In case of one pig (Pig #15), the fever was not recorded at all, even during viremic days. 

The minimum incubation period was estimated as five days, maximum as 17 days with an average of 12 (± 4) days. The average lifespan with viremia was estimated as 4 (± 2) days (Figure 2). Surprisingly, in this group, the mortality rate was 100% within 21 dpi.

**Figure 3:**
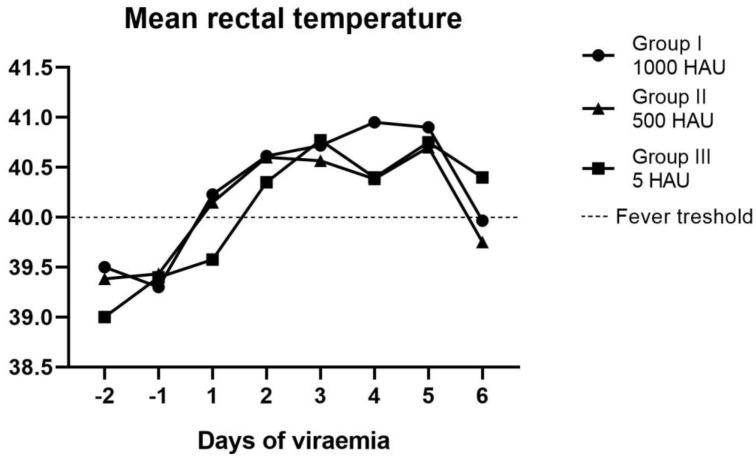
Mean rectal temperatures in the following six days of the detected viremia within the groups.

The mean time interval between viremia and fever (incubation period) was estimated as 1 (± 1) day within Groups II and III, while in Group I, the fever was mostly correlated with viremia. It is noteworthy that, in some cases, fever was delayed significantly (i.e., Pig #14 and Pig #21—4 days) or, in one case, was not detected at all (Pig #15). In the case of two animals (Pig #6 and Pig #10), hypothermia was recorded, and pigs were euthanized. The mean rectal temperature recorded during the experiment is shown in Figure 3.

### 2.2. Clinical Score

**Figure 4:**
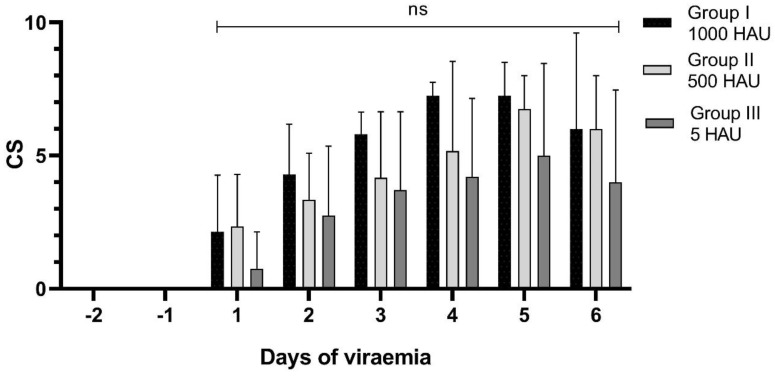
Average clinical scores recorded within groups in the following six days of detected viremia; CS—clinical score, ns—not significant.

A maximum of 10 clinical scores were observed in the case of Pig #8 (a chronic form of the disease), in contrast to Pig #15, where a total of 0 points were estimated (lack of fever and other clinical signs). To ensure comparability between all animals in the groups, an average clinical score (± SD) is presented in the following six days after the detected viremia (Figure 4.) 

### 2.3. Virus Shedding and Load

In rectal samples, the genetic material of the virus was detected within average 2 (± 1) days after detection of the viremia for Group I and within 1 (± 1) day for Groups II and III. The average timespan between detection of the viral DNA in blood and oral fluid samples was estimated at 3 (± 3) days for Group I and 2 (± 1) days for Groups II and III. The estimated latent periods for rectal and oral fluid samples were slightly longer than the timespan of viremia detection (Figure 2) (Table 1). 

**Figure 5:**
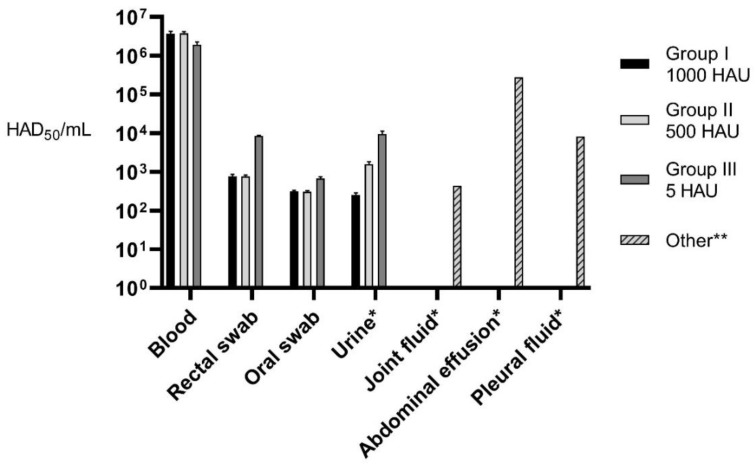
Average maximum virus load in different specimens; *—post mortem **—in selected animals.

**Figure 6:**
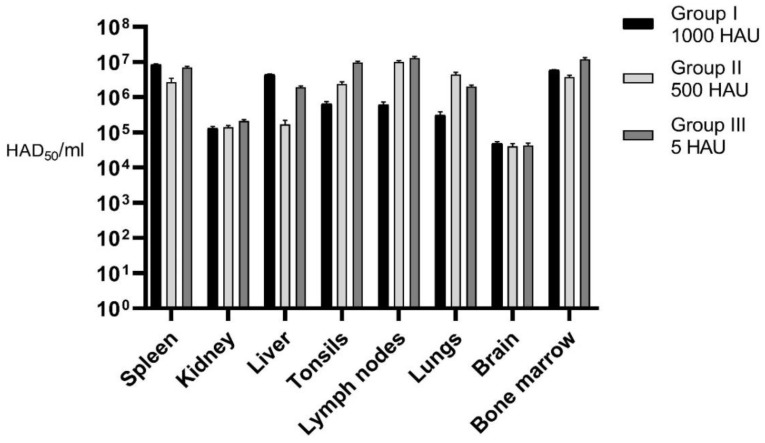
Average virus load in internal organs.

Maximum average (for each Group) virus load in blood, rectal, oral, and urine samples as well as in internal organs after the necropsy was estimated. Additionally, virus load was assessed in such specimens as abdominal effusion, pleural, and joint fluid for selected animals (Figure 5 and Figure 6).

### 2.4. Necropsy

In all groups, similar lesions caused by the disease have been found, except pigs with the chronic form of ASF, in which no pathological lesions (only joint swelling—Pig #8 and enlargement of submandibular lymph nodes—Pig #14) were found.

**Figure 7:**
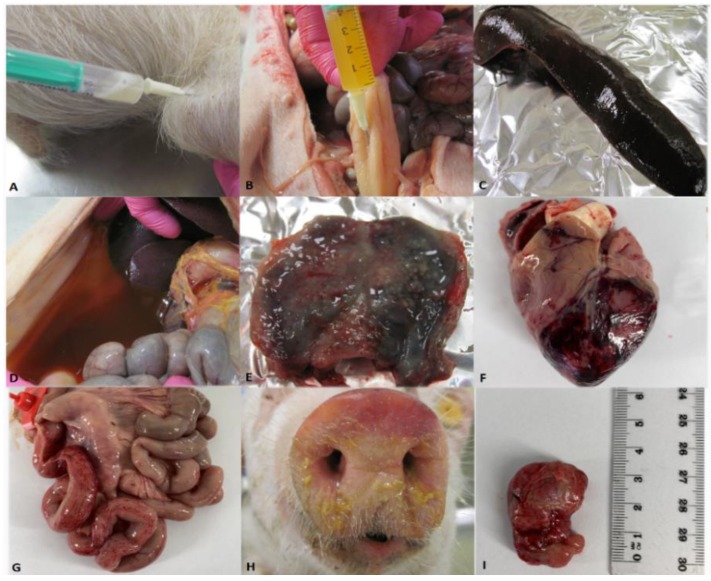
Sample collection: A—joint fluid, B—urine. Post-mortem lesions: C—splenomegaly, D— exudative abdomen fluid, E—hemorrhagic tonsil, F—hemorrhagic heart’s left ventricle, G—intestinal petechiae and enlarged mesenteric lymph nodes, H—nasal discharge, I—enlarged submandibular lymph nodes.

Dark-colored, enlarged spleen (splenomegaly), exudative abdomen fluid, enlarged and hyperemic lymph nodes (submandibular, hepato-gastric, mesenteric), hyperemia of tonsil were most frequently observed lesions (Table 2). In addition, other lesions such as: petechiae in kidneys and intestines, nasal discharge, hemorrhages of lung and heart, pleural, and pericardial exudative fluid were noticed (Figure 7). 

### 2.5. Virus Detection and Isolation

First days of detection, maximum, mean, and minimal Cq values in blood, urine, oral and rectal samples are presented in the Supplementary Table (Table S1). 

The presence of the genetic material of ASFV was confirmed in all the examined blood samples starting from the first day of viremia. Viral DNA was detected in rectal samples (in some cases once) in all animals. It should be mentioned that in the case of four pigs, the DNA of the virus was not detected in oral swab samples.

With regard to the necropsy samples, most of them were positive for ASFV DNA. The genetic material of ASFV was not detected in the spleen, liver, bone marrow, and brain of Pig #8 (the chronic form of ASF). 

The infectious virus was isolated in vitro in the case of most blood, or internal organ samples in the acute or subacute form of the disease, and the hemadsorption phenomenon was observed within 72 hours after the inoculation of the pig with primary alveolar macrophages cell culture (PPAM). For unknown reasons, the virus from oral and rectal samples, as well as in the case of internal organs originating from the pig with the chronic form of ASF (Pig #8) did not show replication in vitro. Moreover, replication of ASFV was excluded in samples showing a lack of hemadsorption phenomenon by qPCR. Viral DNA was undetectable or showed lower concentration in the second passage of hemadsorption-negative samples in comparison to samples from passage 0.

## 3. Discussion 

During this experiment, the disease developed into at least three forms of ASF (acute, subacute, and chronic). Some of the studies published before suggest that the form of the disease may depend on the virulence of a particular virus isolate (i.e., chronic form—low virulent strains, subacute—moderately virulent strains, etc.) [11]. Other studies revealed that the clinical course of ASF might depend not only on the isolate virulence but also could be related to dose, routes of infection, and individual predisposition of animals [12,13,14]. Nevertheless, our study proved that the same virus isolate might cause various clinical forms of the disease. 

The doses used to commence this experiment was sufficient to start infection within all the groups. It stays comparable with other animal trials, where low doses of ASFV were able to infect animals [8,12,15]. There were no statistically significant differences between the groups since the clinical signs and post-mortem lesions were very comparable. However, a few differences, like higher virus load in the internal organs (lymph nodes and tonsils) or delayed fever in the case of Groups with lower doses were noticed. In the light of other studies, it is very likely that in the case of some examined animals, the delayed incubation periods may be consequences of the lack of infection by nasal inoculation. The pathogen was probably administered by direct contact or other routes [13]. Due to the above mentioned, the first day of the detected viremia was applied as a point of reference to retrace the course of the disease and compare it between animals and Groups.

The shortest incubation period was estimated in all three groups at 5 days, and it well corresponds to the previous study (i.e., Latvian isolate) [16]. Pigs with the chronic form of the disease (survivors) presented delayed incubation periods (Pig #8–12 days and Pig #14–20 days). On the other hand, several pigs with the subacute form of ASF also presented delayed incubation periods (i.e., Pig #1, Pig #18–16 days, Pig #21–17 days), thus incubation periods could not be clearly associated with the form of the disease. 

The clinical signs observed in this study were comparable to these described by other authors and were not specific [7,12,17]. Several clinical findings, like cyanosis or hemorrhages of the skin and ears or bloody diarrhea indicated by other authors, were not observed during this experiment. Similarly to other studies, one pig remained clinically healthy, with virus DNA detectable in blood and lesions observed in internal organs by necropsy [7,13]. 

The necropsy lesions observed during this experiment stand in line with these observed previously by other authors; however, the frequency of the observed lesions still deserves attention [7,18,19]. Hyperemia together with the enlargement of lymph nodes and splenomegaly, may be the first lesions, which may facilitate the rapid veterinary diagnosis. In addition, it should be borne in mind that animals with the chronic form of ASF may not present any lesions. 

Despite the fact that isolation of the infectious virus coming from rectal and oral samples was not successful, the probability of infection through saliva or feces cannot be excluded, especially when the survival of the virus in the feces was confirmed and aerosol or direct contact routes of the infection were proved previously by other authors [13,20]. Moreover, an estimated virus load in (diluted) rectal or oral samples almost reached the highest infectious dose (nearly 10^3^ HAD_50_/ml). The carcasses of pigs, blood, and internal organs together with other (i.e., urine, abdominal exudative fluid, joint fluid) materials presented the highest virus load and should be established as the highest risk materials for ensuring biosecurity. 

## 4. Materials and Methods.

### 4.1. Experimental Conditions

A total of twenty-eight domestic pigs, aged from five to six weeks, both sexes, were randomly divided into four Groups—(Group I—ASFV genotype II Pol18_28298_O111, 1000 HAU (n = 8), Group II—ASFV genotype II Pol18_28298_O111, 500 HAU (n = 6), Group III—ASFV genotype II Pol18_28298_O111, 5 HAU (n = 8) and control Group (n = 6)). All animals have been purchased in a commercial local pig farms, with a confirmed high level of health status—serologically free from Porcine reproductive and respiratory syndrome virus (PRRSV), Aujeszky’s disease (PRV) and *Mycoplasma hyopneumoniae*. The Groups and pig numbers are presented in the Supplementary Table (Table S1).

The animals were acclimatized for at least seven days in BSL3 (Biosafety Level-3) animal facility in four independent units with permanent access to feed and water. After the acclimatization phase, at the beginning of the study, the health status of all piglets was evaluated by veterinary examination and confirmed to be free of ASFV by using VIRTOTYPE® Real-time PCR kit (Qiagen, Hilden, Germany).

The animal experiment was approved by the Local Ethical Commission for the Animal Experiments in Lublin (the approval number: 145/2018). All procedures, including euthanasia, were done according to actual law regulations.

### 4.2. Virus and Infection

Intranasal infection using ASFV genotype II Pol18_28298_O111 was conducted with the dose of 1000 HAU to infect animals in Group I and the dose of 500 HAU and 5 HAU to infect animals in the Group II, and Group III respectively.

The virus isolate was derived from the spleen of the naturally infected pig in Poland during the 111 outbreak, which took place in Chełm district (Eastern Poland) in May 2018. The virus was propagated on primary pig alveolar macrophages (PPAM) in RPMI medium (Gibco, Thermo Fisher Scientific, Waltham, USA) supplemented with 10% fetal bovine serum (FBS). 

### 4.3. Clinical Score

Evaluation of rectal temperatures and clinical signs was performed daily and scored accordingly to the clinical score scale, previously applied by several authors, with the slightly modified pointing of fever (rectal temperature < 40 °C—0 points)—for a better representation of the first days of fever during the experiment [12,13]. 

### 4.4. Samples

#### 4.4.1. Rectal and Oral Swabs

Rectal and oral swabs were collected daily to asses virus load and shedding. After sample collection, swabs were placed into tubes containing 4 mL of phosphate-buffered saline (PBS), then incubated in RT for 10 minutes and vortexed. An aliquot of 200 µL of each sample was used for DNA extraction, with a QIAamp DNA Mini Kit, according to the manufacturer’s protocols. The remaining liquid was filtered (0.45 µm) and immediately frozen at −70 °C to assess the titer of the virus. 

#### 4.4.2. Blood 

Blood was collected to plastic tubes containing K2-EDTA, at the beginning at –7,0,1,4 dpi (day post-infection), then at least two times a week or daily, each time when clinical signs (i.e., fever) were recorded. Blood diluted 1:10 in PBS (v/v) was intended for DNA extraction.

#### 4.4.3. Necropsy Samples

The complete necropsy was done on each animal as soon as possible after death or euthanasia. Tissue samples (i.e., the spleen, liver, kidneys, lungs, submandibular lymph nodes, tonsils, brain, and bone marrow) were collected to 50 mL tubes. About 10% dilution in PBS (w/v) of each tissue was done by homogenization in TissueLyser® (Qiagen, Hilden, Germany). In addition, other samples i.e., abdominal exudative fluid, joint fluid and urine were collected during necropsy. A total of 200 µL of each sample was used for DNA extraction and intended for further real-time PCR analysis. 

### 4.5. DNA Detection

Manual column extraction was performed according to Qiagen DNA Mini Kit protocol (Qiagen, Hilden, Germany). Real-time PCR was conducted accordingly to VIROTYPE® (Qiagen, Hilden, Germany) manufacturer’s manual using the Rotor-Gene® Q thermocycler (Qiagen, Hilden, Germany).

### 4.6. Infectious virus Isolation

For virus isolation, PCR-positive samples were designated. Infectious virus in swab samples, blood, and chosen internal organ homogenates were detected by hemadsorption assay in 96-well plates. An aliquot of 50 µL of each filtrated sample (0.45 µm Sartorius syringe filters, Getynga, Germany) was added (in three replicates) to 200 µL growth medium containing: RPMI (Gibco, Waltham, USA), 10% of fetal bovine serum (Gibco, Waltham, USA), 1% of Antibiotic/Antimycotic solution (Sigma Aldrich, St. Louis, USA) and 1:300 (v/v) swine red blood cells (RBC). Hemadsorption phenomenon was observed within five days post-inoculation. All samples negative in hemadsorption assay were passaged twice and tested by qPCR to confirm or exclude virus replication.

### 4.7. Virus Load Estimation

Determination of 50% endpoint titer (HAD_50_/mL) in the selected spleen homogenate (originated from the experiment) was assumed in pig alveolar macrophages (DTU, Danemark) using Spearman–Karber method. A series of 10-fold dilutions (in three replicates) of tittered spleen homogenates were prepared in phosphate-buffered saline (PBS), and the Cq value of each dilution was estimated by Real-time PCR (VIROTYPE®, Qiagen, Hilden, Germany). A standard curve was prepared (Microsoft Excel, Windows), and relevant Cq values obtained during the experiment were estimated and expressed as virus titer.

### 4.8. Statistical Analysis

For all Groups, the analysis was done on different days of the detected viremia, determined as the period from the first day of the detected viremia to death or euthanasia. The analyzed data were compared between the groups using a one-way analysis of variance (one-way ANOVA) in GraphPad Prism (GraphPad Software, San Diego, USA).

## 5. Conclusions

These studies give an overview of Veterinary Inspection of the principles of proper clinical assessment of different forms of ASF. Low doses (5 HAU) of ASFV may develop the infection. In the course of ASF caused by Pol_28298_O111 isolate, clinical signs are not specific; the fever may occur a few days after viremia or may not be detectable at all. Shortly after viremia, the virus is secreted with feces and saliva in the amounts which may be a potential source of infection. Moreover, in some cases, there were no observable abnormalities with feed intake among the infected animals. In relation to these observations, the Veterinary Inspection, especially in large pig farms, may have difficulties in ASF diagnosis since no obvious or specific clinical sings could be observed. 

Therefore, each case of unexplained animal death in pig farms (especially these located in risk areas) should be immediately diagnosed and the carcass (as the material with the highest viral load) properly disposed and quickly utilized, in accordance with biosecurity procedures. Each time detection of the disease should be confirmed by real-time PCR, by examining properly chosen samples (i.e., blood) to ensure a high level of detection. 

## Figures and Tables

**Table 1:** Estimated average (± SD) latent periods for blood, rectal, and oral fluid samples, and average time interval between the detection the virus in the blood and detection in oral and rectal specimens.

Group	Average Latent Period (± SD) (days)			Average Shedding Timespan between viremia and Samples (± SD) (days)	
	Blood	Rectal	Oral	Rectal	Oral
Group I	9 (± 4)	10 (± 4)	14 (± 4)	2 (± 1)	3 (± 3)
Group II	11 (± 4)	12 (± 3)	13 (± 4)	1 (± 1)	2 (± 1)
Group III	11 (± 3)	12 (± 3)	13 (± 2)	1 (± 1)	2 (± 1)

**Table 2:** Frequency of lesions observed in necropsy. The numbers represent the number of animals with detected lesions. The total number of animals with an observed lesion in relation to all the infected pigs (n = 22) is expressed as the total frequency.

Lesion	Group I (n = 8)	Group II (n = 6)	Group III (n = 8)	Total Frequency
Splenomegaly	7	3	8	82%
Hyperaemia and/or enlargement of lymph nodes *	6	4	8	82%
Abdomen exudative fluid	4	3	3	45%
Hyperaemia of tonsil	5	3	2	45%
Petechiae in kidneys	1	2	2	23%
Pleural exudative fluid	1	1	2	18%
Hyperaemia of lungs	1	2	1	18%
Nasal discharge	2	2	0	18%
Pericardial exudative fluid	0	1	1	9%
Petechiae in intestines	0	2	0	9%
Petechiae in liver	1	0	0	5%
Heart - haemorrhage	0	0	1	5%

*—submandibular, mesenteric or hepato-gastric.

## Supplementary Materials

The following are available online at https://www.mdpi.com/2076-0817/9/3/237/s1, Table S1: Raw data of individual parameters obtained during the experiment (first days of fever; first days of African swine fever virus (ASFV) DNA detection in blood, oral, and rectal samples; minimal and maximal Cq values obtained for blood, rectal, oral and urine samples).

## References

[1] Sánchez-Cordón P.J., Montoya M., Reis A.L., Dixon L.K. (2018). African swine fever: A re-emerging viral disease threatening the global pig industry. Vet. J..

[2] Juszkiewicz M., Walczak M., Woźniakowski G. (2019). Characteristics of selected active substances used in disinfectants and their virucidal activity against ASFV. J. Vet. Res..

[3] Sánchez E.G., Pérez-Núñez D., Revilla Y. (2019). Development of vaccines against African swine fever virus. Virus Res..

[4] Dixon L.K., Stahl K., Jori F., Vial L., Pfeiffer D.U. (2019). African Swine Fever Epidemiology and Control. Annu. Rev. Anim. Biosci..

[5] Rowlands R.J., Michaud V., Heath L., Hutchings G., Oura C., Vosloo W., Dwarka R., Onashvili T., Albina E., Dixon L.K. (2008). African Swine Fever Virus Isolate, Georgia, 2007. Emerg. Infect. Dis..

[6] Schulz K., Staubach C., Blome S., Viltrop A., Nurmoja I., Conraths F.J., Sauter-Louis C. (2019). Analysis of Estonian surveillance in wild boar suggests a decline in the incidence of African swine fever. Sci. Rep..

[7] Pikalo J., Zani L., Hühr J., Beer M., Blome S. (2019). Pathogenesis of African swine fever in domestic pigs and European wild boar – Lessons learned from recent animal trials. Virus Res..

[8] Pershin A., Shevchenko I., Igolkin A., Zhukov I., Mazloum A., Aronova E., Vlasova N., Shevtsov A. (2019). A long-term study of the biological properties of ASF virus isolates originating from various regions of the russian federation in 2013-2018. Vet. Sci..

[9] Gallardo C., Soler A., Nieto R., Sánchez M.A., Martins C., Pelayo V., Carrascosa A., Revilla Y., Simón A., Briones V. (2015). Experimental Transmission of African Swine Fever (ASF) Low Virulent Isolate NH/P68 by Surviving Pigs. Transbound. Emerg. Dis..

[10] Gallardo C., Arias M. (2019). African swine fever (ASF) diagnosis, an essential tool in the epidemiological investigation. Virus Res..

[11] Sánchez-Vizcaíno J.M., Mur L., Gomez-Villamandos J.C., Carrasco L. (2015). An update on the epidemiology and pathology of African swine fever. J. Comp. Pathol..

[12] Pietschmann J., Guinat C., Beer M., Pronin V., Tauscher K., Petrov A., Keil G., Blome S., Tauscher K., Petrov A. (2015). Course and transmission characteristics of oral low-dose infection of domestic pigs and European wild boar with a Caucasian African swine fever virus isolate. Arch. Virol..

[13] Olesen A.S., Lohse L., Boklund A., Halasa T., Gallardo C., Pejsak Z., Belsham G.J., Bruun T., Bøtner A. (2017). Transmission of African swine fever virus from infected pigs by direct contact and aerosol routes. Vet. Microbiol..

[14] Karalova E., Zakaryan H., Voskanyan H., Hakobyan A., Nersisyan N., Saroyan D., Karalyan N., Tatoyan M., Akopian J., Gazaryantz M. (2015). Clinical and post-mortem investigations of genotype II induced African swine fever. Porc. Res..

[15] Niederwerder M.C., Stoian A.M.M., Rowland R.R.R., Dritz S.S., Petrovan V., Constance L.A., Gebhardt J.T., Olcha M., Jones C.K., Woodworth J.C. (2019). Infectious Dose of African Swine Fever Virus When Consumed Naturally in Liquid or Feed. Emerg. Infect. Dis..

[16] Gallardo C., Soler A., Nieto R., Cano C., Pelayo V., Sanchez M.A., Pridotkas G., Fernandez-Pinero J., Briones V., Arias M. (2017). Experimental Infection of Domestic Pigs with African Swine Fever Virus Lithuania 2014 Genotype II Field Isolate. Transbound. Emerg. Dis..

[17] Petrini S., Feliziani F., Casciari C., Giammarioli M., Torresi C., De Mia G.M. (2019). Survival of African swine fever virus (ASFV) in various traditional Italian dry-cured meat products. Prev. Vet. Med..

[18] Zani L., Forth J.H., Forth L., Nurmoja I., Leidenberger S., Henke J., Carlson J., Breidenstein C., Viltrop A., Höper D. (2018). Deletion at the 5’-end of Estonian ASFV strains associated with an attenuated phenotype. Sci. Rep..

[19] Sánchez-Cordón P.J., Nunez A., Neimanis A., Wikström-Lassa E., Montoya M., Crooke H., Gavier-Widén D. (2019). African swine fever: Disease dynamics in wild boar experimentally infected with ASFV isolates belonging to genotype I and II. Viruses.

[20] Davies K., Goatley L.C., Guinat C., Netherton C.L., Gubbins S., Dixon L.K., Reis A.L. (2017). Survival of African Swine Fever Virus in Excretions from Pigs Experimentally Infected with the Georgia 2007/1 Isolate. Transbound. Emerg. Dis..

