# Supplementary material for: Analysis of the Clinical Course of Experimental Infection with Highly Pathogenic African Swine Fever Strain, Isolated from an Outbreak in Poland. Aspects Related to the Disease Suspicion at the Farm Level
Source: Pathogens. 2020 Mar 22;9(3):237. doi: 10.3390/pathogens9030237 (PMC7157746; doi:10.3390/pathogens9030237)
Supplement: Supplementary file 1 [file pathogens-09-00237-s001.pdf]

Table S1. Raw data of individual parameters obtained during experiment.

Group	Pig number	F (dpi)	A (dpi)	Blood (Cq)			B (dpi)	Rectal (Cq)			C (dpi)	Oral (Cq)			Urine (Cq)**
				min	mean(±SD)	max		min	mean(±SD)	max		min	mean(±SD)	max	
Group I 1000 HAU	1	16	16	19.4	23.4(5.7)	34.5	18	30.7	34.3(3.5)	38.9	16	31.5	35.9(2.8)	38.9	35.0
	2	4	4	20	22.8 (3.3)	27.4	5	34.3	35(0.7)	35.7	n/d	n/a	n/a	n/a	38.6
	3	8	8	20.1	27.3(7.3)	37.4	10	38.5*	38.5(0)*	38.5	9	39.4*	39.4(0)*	39.4*	29.9
	4	5	4	19.6	21(1.4)	22.4	5	35.8*	35.8(0)*	35.8	n/d	n/a	n/a	n/a	27.3
	5	6	n/d	n/a	n/a	n/a	7	39.1*	39.1(0)*	39.1*	n/d	n/a	n/a	n/a	34.1
	6	10	9	20.1	25.3(5.0)	35.2	12	30.3	31.7(2.2)	34.9	12	35.0	37.2(1.9)	39.6	38.5
	7	10	10	19.9	22.0(1.9)	25.4	11	28.3	33.5(3.3)	37.3	11	34.6	35.1(0.5)	36.1	38.2
	8	12	12	29.4	32.3 (1.7)	34.6	15	27.4	31.9(3.4)	37.6	20	32.8	35.9(1.9)	38.1	38.6
Group II 500 HAU	9	12	12	23.4	25.0(2.7)	29.5	14	30.9*	30.9(0)*	30.9*	14	32.6	33.5(1.2)	34.4	33.5
	10	11	11	16.2	20.3(3.1)	26.2	13	29.4	30.6(0.8)	31.6	12	27.8	31.3(2.9)	34.8	19.1
	11	14	13	23.5	27.1(4.4)	36.1	14	28.4	29.1(0.7)	34.2	16	35.4	35.9(0.5)	36.5	n/d
	12	5	5	19.7	21.6(2.5)	26.4	7	26.6	29.6(2.8)	33.6	7	31.0	34.5(3.3)	38.7	32.2
	13	6	6	19.3	22.5(2.0)	24.2	8	29.7	32.9(3.2)	36.1	9	39.3	39.3(0)	39.3	31.2
	14	20	16	29.6	31.7(1.4)	34.1	16	29.9	35.4(2.5)	37.2	18	33.6	35.0(1.3)	37.6	n/a
Group III 5 HAU	15	n/d	9	18.7	23.9(5.2)	31.1	11	37.0*	37.0(0)*	37.0*	n/d	n/a	n/a	n/a	32.6
	16	12	11	24.4	27.0(2.3)	32.0	13	29.6	31.1(1.5)	33.2	14	33.9	34.9(1)	35.9	n/d
	17	11	11	19.3	20.3(1.0)	21.3	12	34.4	34.4(0)*	34.4	12	37.2*	37.2(0)*	37.2*	24.7
	18	16	14	24.8	29.2(4.9)	37.8	14	34.7	37.2(1.4)	38.2	14	30.3	34.1(3.1)	38.9	34.6
	19	12	11	20.8	21.4(1.2)	23.1	13	34.3*	34.3(0)*	34.3*	12	38.3	38.4(0.1)	38.4	25.6
	20	5	4	18.8	22.2(2.5)	25.9	5	32.9	34.9(2.7)	38.7	7	34.3	34.3(0)	34.3	38.8
	21	17	13	20.1	27.9(5.1)	36.5	15	30.2	35.0(2.6)	38.0	14	33.5	35.8(1.75)	38.3	34.8
	22	13	13	22.5	23.2(0.6)	23.9	13	31.3	33.1(1.4)	34.8	15	35.2	36.4(1.2)	37.6	n/a

F- first fever day (incubation period); First day of detection in: A – blood; B - rectal swabs; C - oral swab; *- single detection ** - post mortem; n/a – not applicable n/d – not detected
